# Carotid Beta Stiffness Association with Thyroid Function

**DOI:** 10.3390/jcm10030420

**Published:** 2021-01-22

**Authors:** Alessandro P. Delitala, Angelo Scuteri, Edoardo Fiorillo, Valeria Orrù, Edward G. Lakatta, David Schlessinger, Francesco Cucca

**Affiliations:** 1 Department of Medical, Surgical, and Experimental Sciences, University of Sassari, 07100 Sassari, Italy; ascuteri@uniss.it; 2 Istituto di Ricerca Genetica e Biomedica (IRGB), Consiglio Nazionale delle Ricerche, c/o Cittadella Universitaria di Monserrato, 09042 Cagliari, Italy; edoardo.fiorillo@irgb.cnr.it (E.F.); valeria.orru@irgb.cnr.it (V.O.); fcucca@uniss.it (F.C.); 3 Laboratory of Cardiovascular Sciences, National Institute on Aging Intramural Research Program, National Institutes of Health, Baltimore, MD 21224, USA; lakattae@grc.nia.nih.gov; 4 National Institute on Aging, NIH, DHHS, Baltimore, MD 21224, USA; SchlessingerD@grc.nia.nih.gov; 5 Department of Biomedical Sciences, University of Sassari, 07100 Sassari, Italy

**Keywords:** thyroxine, carotid beta stiffness, aging, carotid strain, thyroid hormone, heart rate

## Abstract

Background: Thyroid hormone modulation of cardiovascular function has been associated with cardiovascular disease. Recent evidence suggests that free thyroxine (FT4) levels are associated with an increase in systemic arterial stiffness, but little is known about the effects of FT4 at the local level of the common carotid artery. β-stiffness index is a local elastic parameter usually determined by carotid ultrasound imaging. Methods: We conducted a cross-sectional analysis in the ProgeNIA cohort, including 4846 subjects across a broad age range. For the purpose of this study, we excluded subjects with increased thyrotropin (TSH) levels and those treated with levothyroxine or thyrostatic. We assessed β stiffness, strain, wall–lumen ratio, carotid cross-sectional area (CSA), and stress and flow in the right common carotid artery. We tested whether FT4, heart rate, and their interactions were associated with carotid parameters. Results: FT4 was positively and independently associated with β stiffness index (β = 0.026, *p* = 0.041), and had a negative association with strain (β = −0.025, *p* = 0.009). After adding heart rate and the interaction between FT4 and heart rate to the model, FT4 was still associated with the β stiffness index (β = 0.186, *p* = 0.06), heart rate was positively associated with the stiffness index (β = 0.389, *p* < 0.001) as well as their interaction (β = 0.271, *p* = 0.007). Conclusion: This study suggests that higher FT4 levels increase arterial stiffness at the common carotid level, consistent with a detrimental effect on elastic arteries. The effect of FT4 is likely to be primarily attributable to its effect on heart rate.

## 1. Introduction

Thyroid hormone physiologically regulates different cardiovascular parameters [1]. These effects become more evident during disorders of the thyroid gland. Hypothyroidism usually has a detrimental effect on lipid metabolism [2], with an increase in atherogenic low density lipoprotein cholesterol (LDL) and triglycerides, along with gender and sex hormone effects having been also postulated [2,3]. The role of levo-thyroxine replacement therapy has been clearly demonstrated in overt disease, but its role in subclinical hypothyroidism remains less clear [4,5]. In contrast to low thyroid function, hyperthyroidism causes an increase in metabolic rate, and is associated with increased cardiovascular activity [6]. Indeed, increased heart contractility, frequency, and vasodilation are typically noted in hyperthyroid patients [7]. 

Although changes associated with aging have not yet been adequately characterized, aging is well known to have profound effects on the cardiovascular system. Arterial stiffening, beginning early in the life-course [8,9], is a major feature of aging and has been associated with increased cardiovascular (CV) morbidity and disability [10]. 

Due to its ability to predict early vascular disease, the evaluation of arterial stiffness has been recommended as a diagnostic tool in the most recent guidelines from the European Society of Cardiology (ESC) [11]. Different techniques have been developed to measure arterial stiffness non-invasively, and carotid-femoral pulse wave velocity (PWV) is acknowledged as the gold standard to assess arterial stiffness [12]. The β stiffness index reflects age-related stiffening of the carotid artery [13]. The resulting impairment causes a loss of the ability to buffer a pulsatile flow and the pressure generated during systole, thus transmitting excessive flow pulsatility and pressure into the brain microcirculation [14,15].

Carotid stiffness has been associated with the incident of stroke [16], incident of CV events [17], and a reduction in renal function [18]. Compared to carotid-femoral PWV, exploring systemic large artery stiffening of the carotid can provide more information on local alterations in the elastic properties of the arterial wall biomaterial [19], on functional arterial stiffening secondary to endothelial dysfunction [20], and local alterations in pulsatility [21], with consequent carotid remodelling [22]. 

The role of thyroid hormone on arterial stiffening is not clear. Indeed, previous studies reported a negative effect of hypothyroidism on PWV [23], which improved after treatment with levothyroxine [24]. However, a previous study by our group demonstrated a positive association between free thyroxine (FT4) and increased carotid-femoral PWV [25]. In this study we extend the analysis, testing the association between thyroid hormones and the β stiffness index at the common carotid level. 

## 2. Materials and Methods

The ProgeNIA study investigates hundreds of genotypic and phenotypic aging-related traits in a longitudinal survey [26,27]. From the initial sample of 6148 individuals, subjects who reported taking thyroid medications (thyroid hormone replacement or thyrostatics) or drugs that alter thyroid function tests (amiodarone, lithium, and corticosteroids), and those with subclinical or overt hypothyroidism (i.e., increased thyrotropin (TSH)) were excluded. Younger participants (i.e., aged < 20 years) were also excluded, yielding a final sample of 4846 (aged 20–97 years). All had routine medical examinations including: (i) measurements of height, weight, systolic blood pressure (SBP) and diastolic blood pressure (DBP); (ii) medical history, including therapy; (iii) blood sampling (see below); and (iv) carotid ultrasound (see below). 

Each participant signed an informed consent form. All study methods were conducted according to the principles expressed in the Declaration of Helsinki and were approved by the governing Ethics Committee, Azienda Sanitaria Locale 4 (ASL4), protocol no. 2171/CE.

### 2.1. Biochemical and Hormone Assays

Venous blood samples were drawn between 7 and 8 a.m. after an overnight fast. Serum samples were stored at −80°C until use. Plasma triglycerides and total cholesterol were determined by an enzymatic method (Abbott Laboratories ABA-200 ATC Biochromatic Analyzer, Irving, TX, USA). High density lipoprotein cholesterol (HDL) was determined by dextran sulphate–magnesium precipitation. Low density lipoprotein (LDL) concentrations were estimated by the Friedewald formula. Fasting plasma glucose concentration was measured by the glucose oxidase method (Beckman Instruments Inc., Fullerton, CA, USA).

TSH and FT4 were assessed with a two-site, solid-phase chemiluminescent immunometric assay, as described elsewhere [28]. Normal values are considered to be in the range TSH, 0.4–4.0 μIU/mL; FT4, 0.89–1.76 ng/dL.

Overt hyperthyroidism was diagnosed in the presence of reduced TSH and FT4, displaying levels over the higher limit of the reference range. We defined subclinical hyperthyroidism as the presence of serum FT4 level in the normal reference range together with low serum TSH. Euthyroidism was defined as the presence of TSH concentration within the reference range. 

### 2.2. Arterial Structure and Function

Carotid ultrasound was performed with a linear-array, 5- to 10-MHz transducer (Ultramark 9 HDI, Advanced Technology Laboratories, Inc., Seattle, WA, USA), as previously described. Briefly, the subject was placed in the supine position with his/her neck in extension and rolled contralaterally by about 45°. The right common carotid artery was examined at 1.5 cm proximal to the carotid bifurcation. Carotid parameters, β stiffness, strain, wall–lumen ratio (W/L), carotid cross-sectional area (CSA), stress and flow, were calculated as previously reported [29].

### 2.3. Definition of Cardiovascular Risk Factors

Hypertension was defined as SBP ≥ 140 mmHg, and/or DBP ≥ 90 mmHg, and/or self-reported use of antihypertensive drugs. Pulse pressure (PP) was defined as SBP—DBP. Body mass index (BMI) was calculated as weight (kg)/height^2^ (m^2^). Diabetes mellitus was defined as self-reported diagnosis of diabetes and/or self-reported use of antidiabetic drugs or elevated fasting glycated haemoglobin or fasting glycaemia, according to the American Diabetes Association guidelines [30]. Dyslipidaemia was defined as self-reported use of lipid-lowering medications or the finding of LDL levels ≥ 140 mg/dL. Smokers were defined as current consumers of at least one cigarette per day. We defined the term “cardiovascular event” as the documented history of myocardial infarction or stroke.

Heart rate was determined by electrocardiography, recorded before collecting ultrasound scan of the carotid artery.

### 2.4. Statistical Analysis 

Since continuous variables were not distributed normally (Shapiro–Wilk test), nonparametric tests (Wilcoxon rank-sum test, Kruskal–Wallis test and Spearman’s correlation) were used for the univariate analysis. Mann–Whitney U test was used to compare groups of thyroid function, considering euthyroid as a control. To assess the relationship between dependent variables (parameters of carotid structure) and thyroid hormones (TSH and FT4), we used a stepwise regression analysis including all traits significantly associated with dependent variables. Although associated, SBD and DBP were not further included in the analyses due to the presence of collinearity. The final model included age, sex, BMI, PP, previous CV events, presence of hypertension, heart rate and FT4. To test the interaction terms, heart rate-by-FT4 was entered in the multilevel model. Tests of significance were two-sided, and a *p* < 0.05 was assumed as statistically significant in Stata 12 for Mac. 

## 3. Results

Descriptive analyses are presented in Table 1, stratified by categories of thyroid function. Subjects with subclinical hyperthyroidism were older, had higher blood pressure values and levels of glycemia (*p* < 0.001 for all), and lower concentrations of triglycerides (*p* < 0.05) as compared to euthyroidism. In addition, participants with subclinical hyperthyroidism had higher BMI (*p* < 0.001), and HDL (*p* < 0.005) values in comparison to euthyroid individuals. Subjects with overt hyperthyroidism had a higher PP and DBP (*p* < 0.05) and heart rate (*p* < 0.001). Frequency of diabetes and hypertension was increased in subclinical hyperthyroidism. 

Table 2 shows cardiovascular parameters at the right common carotid level. Subjects with subclinical hyperthyroidism had a higher stiffness index, circumferential stress area (CSA), and circumferential stress and lower strain (*p* < 0.001 for all). Subjects with overt hyperthyroidism had higher wall–lumen ratios as compared to euthyroid, although this was not statistically significant.

Table 3 shows the effects of FT4 and heart rate on parameters of common carotid structure. FT4 showed a positive and significant association with the β stiffness index (β = 0.026, *p* < 0.05) and an inverse association with strain (β = −0.025, *p* < 0.05). After adding heart rate and the interaction term between FT4 and heart rate (FT4-by-heart rate) to the model, FT4 was still associated with β stiffness index (β = 0.186, *p* = 0.06), heart rate was positively associated with stiffness index (β = 0.389, *p* < 0.001) and the interaction between the two (β = 0.271, *p* = 0.007). 

TSH was not associated with any of the carotid parameters. 

## 4. Discussion

We report a primary finding of the association between thyroid hormone and carotid parameters at the common carotid level, showing a positive association of FT4 with local arterial stiffening. 

The role of thyroid hormone in the cardiovascular system has long been clear, with both hypothyroidism and hyperthyroidism associated with detrimental effects. Hypothyroidism leads to a worse lipid profile, with increased total cholesterol, LDL and triglyceride levels, while hyperthyroidism increases the risk of cardiac arrhythmias, heart failure, and ischemic stroke, especially in elderly patients. While these associations are clearly present in case of overt dysfunctions, there is no agreement about their effects in subclinical disorders [7,31]. 

The stiffening of arteries can be easily measured with carotid-femoral PWV, which is an independent risk factor for CV disease, all-cause mortality, and CV mortality. Some reports have found additional associations with mortality in type 2 diabetes [32] and end-stage renal disease [33]. However, the carotid-femoral PWV reflects the structural properties of the aorta, including elastic and muscular features. By contrast, the carotid is mostly elastic in nature, and its stiffness predicts the incident of stroke independently of other CV risk factors and aortic stiffness [34]. Thus, it is important to measure arterial stiffness at different sites along the non-uniform arterial tree. 

Our analyses show that stiffness was increased in subjects with subclinical and overt hyperthyroidism, as shown in Table 2. However, the univariate analysis reported a non-progressive increase along with increasing values of FT4, which was probably due to the higher age of the subjects with subclinical hyperthyroidism. Indeed—and indicated clearly by the multivariate analysis, even after adjusting for different cardiovascular variables—FT4 has a positive and direct association with stiffness. Clinical conditions characterized by increased concentrations of thyroid hormone, even if mild, can have a detrimental effect on the CV system. Indeed, subclinical hyperthyroidism has been reported as being associated with an increased risk of ischemic stroke in different studies, mainly due to a “trigger effect” on the onset of atrial fibrillation, though other studies failed to find such an association. Because carotid stiffening may lead to rupture of atherosclerotic carotid plaques [35], it can be speculated that an excess of thyroid hormone can increase the risk of stroke through two different mechanisms: by increasing the risk of atrial fibrillation and by increasing stiffness at the common carotid level. This hypothesis needs to be tested with specific studies, which are not feasible in this study. One can also suggest that atherosclerosis is more common in patients with subclinical hypothyroidism due to the more atherogenic lipid profile. However, it should be noted that some studies failed to report an increased frequency of atherosclerotic plaque in patients with subclinical hypothyroidism, as compared to euthyroid and subclinical hyperthyroidism patients [36]. Understanding the impact of thyroid disorders on atherosclerosis is further complicated by the findings of Volzke et al., who reported increased intima-media thickness in patients with subclinical hyperthyroidism [37]. 

The relationship between thyroid hormone and carotid stiffness is, thus, intricate and studies in the literature are inconclusive. Tatar et al. reported an inverse association between carotid stiffness and free triiodothyronine in a sample of 137 patients with end-stage renal disease on hemodialysis [38]. The same group reported similar results in euthyroid patients on peritoneal dialysis [39]. Another study found increased stiffness at the common carotid level in 46 hypothyroid individuals compared to healthy controls [40]. Treatment with levo-thyroxine reversed the arterial stiffness, apart from the thickening of the artery wall [41]. On the other hand, consistent with our results, stiffness at the common carotid level has been reported to be increased in hyperthyroid patients with Graves’ disease [42]. 

The interpretation of these divergent results is not clear, but it should be taken into account that the samples in these studies were small, and statistical analyses were not always adjusted for cardiovascular risk factors. By contrast, our multivariate analysis, adjusted for age, sex, BMI, PP, hypertension, and CV events, showed that FT4 had a direct effect on carotid β stiffness. An additional finding of our study is the interaction between FT4 and heart rate. An elevated resting heart rate is associated with CV events, CV death and all-cause mortality [43]. In addition, it has been reported that a higher resting heart rate itself is associated with increased arterial stiffness, with a stronger association observed for the carotid artery compared with the central artery [44]. Thus, a likely explanation of our findings is that FT4 increased carotid stiffness by increasing heart rate. 

Accordingly, thyroid hormones decrease systemic vascular resistance by vasodilation of the peripheral circulation [45], possibly as a direct action on vascular smooth muscle cells or through stimulation of the levels of vasodilatory endothelium-derived substances such as nitric oxide. Thus, a higher FT4 level could result in higher levels of triiodothyronine, thus affecting peripheral vasodilation and reflex cardiac effects such as increases in stroke volume and heart rate. A direct thyroid effect enhancing sinoatrial node activity could also contribute to the increase in heart rate [46]. However, other possible mechanisms cannot be excluded at present. For example, increased total artery stiffness may be a dynamic adaptation to the hypermetabolic state, promoting blood distribution into the peripheral vascular system.

In summary, we found an association between FT4 and arterial stiffness at the common carotid level. In line with the findings of this study, we previously showed that FT4 has a direct positive association with carotid-femoral PWV [25]. This suggests that higher FT4 levels have a detrimental effect on both mixed elastic and muscular arteries, as well as on the more prevalent elastic arteries such as the carotid artery. The effect of FT4 is likely attributable to the effect of FT4 on heart rate.

## Figures and Tables

**Table 1:** Descriptive characteristics of the sample.

	Overt Hyperthyroidism	Subclinical Hyperthyroidism	Euthyroidism	Total
n	24	136	4686	4846
Age, yrs	44.8 (31.3–64.4)	57.7 (45.8–67.3) *	43.2 (32.2–57.5)	43.5 (32.5–58.0)
BMI, Kg/m^2^	26.7 (22.6–29.9)	26.6 (23.8–29.1) *	25.1 (22.3–28.2)	25.2 (22.3–28.3)
Glycemia, mg/dL	85 (79–94)	90 (82–101) *	86 (79–94)	86 (79–94)
Total cholesterol, mg/dL	202 (168–232)	212 (186–244)	211 (184–239)	211 (184–239)
LDL, mg/dL	119 (102–142)	130 (111–155)	128 (105–151)	128 (106–151)
HDL, mg/dL	61 (52–73)	65 (56–74) ^#^	63 (54–73)	63 (54–73)
Triglycerides, mg/dL	68 (51–96)	63 (48–88) ^#^	74 (52–108)	73 (52–107)
SBP	125 (112–140)	130 (119–145) *	123 (113–137)	123 (113–137)
DBP	70 (67–80) ^##^	80 (73–86) *	77 (70–85)	77 (70–85)
PP	51 (43–62) ^##^	50 (41–61) *	47 (40–55)	47 (40–55)
Heart rate	76 (71–87) **	67 (60–76)	66 (59–74)	66 (59–74)
TSH, mUI/mL	0.11 (0.01–0.04) **	0.24 (0.11–0.33) *	1.56 (1.04–2.17)	1.52 (1.00–2.15)
FT4, ng/mL	2.04 (1.90–2.35) **	1.33 (1.18–1.46)	1.28 (1.17–1.41)	1.29 (1.17–1.41)
Female, n (%)	16 (66.7%)	85 (62.5%)	2580 (55.1%)	2681 (55.3%)
Smoker, n (%)	8 (33.3%)	16 (11.8%) ^#^	986 (21.4%)	1010 (20.8%)
CV event, n (%)	0 (0.0%)	3 (2.2%)	71 (1.5%)	74 (1.5%)
Diabetes, n (%)	2 (8.3%)	15 (11.0%) *	211 (4.5%)	228 (4.7%)
Dyslipidemia, n (%)	2 (8.3%)	32 (22.1%)	971 (20.7%)	1005 (20.7%)
Hypertension, n (%)	8 (33.3%)	58 (42.7%) *	1396 (29.8%)	1462 (30.2%)

BMI, body mass index; LDL, low density lipoprotein cholesterol; HDL, high density lipoprotein cholesterol; SBP, systolic blood pressure; DBP, diastolic blood pressure; PP, pulse pressure; TSH, thyrotropin, FT4, free thyroxine, CV, cardiovascular. * Subclinical hyperthyroidism vs. euthyroidism, *p* < 0.001; ^#^ Subclinical hyperthyroidism vs. euthyroidism, *p* < 0.05; ** Overt hyperthyroidism vs. euthyroidism, *p* < 0.001; ^##^ Overt hyperthyroidism vs. euthyroidism, *p* < 0.05.

**Table 2:** Effect of thyroid status on common carotid structure.

	Overt Hyperthyroidism	Subclinical Hyperthyroidism	Euthyroidism	Total
CCA stiffness	6.87 (4.78–9.10)	6.88 (5.54–8.96) *	5.78 (4.58–7.66)	5.81 (4.60–7.70)
CCA strain, %	8.51 (7.55–13.0)	7.94 (6.00–10.4) *	8.93 (6.82–11.3)	8.89 (6.78–11.3)
CCA CSA	21.5 (18.8–24.6)	22.0 (18.6–27.4) *	19.6 (16.9–23.7)	19.6 (17.0–23.8)
CCA W/L	0.215 (0.175–0.244)	0.215 (0.196–0.240) *	0.200 (0.177–0.223)	0.200 (0.178–0.224)
CCA stress	19.9 (15.5–21.9)	20.8 (17.8–24.8) *	18.3 (15.8–21.6)	18.4 (15.8–21.7)

CCA, common carotid artery; CSA, circumferential stress area; W/L, wall to lumen ratio. * Subclinical hyperthyroidism vs. euthyroidism, *p* < 0.001.

**Table 3:** Predictors of parameters of common carotid structure.

		Beta Stiffness *	Strain *	W/L *	CSA *	Stress *
**Model a**						
FT4	β	0.026	−0.025	0.010	0.010	0.013
	*p* value	0.041	0.048	0.472	0.319	0.303
**Model b**						
FT4	β	0.186	−0.109	0.060	0.018	0.072
	*p* value	0.006	0.103	0.438	0.748	0.305
Heart rate	β	0.389	−0.342	−0.001	−0.143	0.031
	*p* value	<0.001	<0.001	0.992	0.812	0.679
FT4-by-heart rate	β	0.271	0.160	−0.070	−0.001	0.086
	*p* value	0.007	0.113	0.550	0.913	0.415

* Adjusted for age, sex, body mass index, pulse pressure, previous cardiovascular events.

## Data Availability

The data presented in this study are available on request from the corresponding author.
